# Association of Metformin Use with Asthma Exacerbation in Patients with Concurrent Asthma and Diabetes: A Systematic Review and Meta-Analysis of Observational Studies

**DOI:** 10.1155/2020/9705604

**Published:** 2020-08-04

**Authors:** Li Wen, Wang Zhong, Yihui Chai, Qin Zhong, Jie Gao, Liancheng Guan, Zhang mengzhi, Liu Huaiquan, Yu Haiyang, Wang Qingxue, Yang Changfu, Chen Yunzhi

**Affiliations:** ^1^School of Preclinical Medicine, Guizhou University of Traditional Chinese Medicine, Guiyang, Guizhou, China; ^2^Department of Clinical Nutrition, Chengdu Fifth People's Hospital, Chengdu, Sichuan, China; ^3^Second Affiliated Hospital, Guizhou University of Traditional Chinese Medicine, Guiyang, Guizhou, China; ^4^Ethnic Medical Center, Guizhou University of Traditional Chinese Medicine, Guiyang, Guizhou, China

## Abstract

**Background:**

Asthma and diabetes are both diseases that affect a wide range of people worldwide. As a common treatment for diabetes, metformin has also been reported to be effective in improving asthma outcomes. We conducted a combined analysis to examine the efficacy of metformin in reducing asthma exacerbation in patients with concurrent asthma and diabetes.

**Methods:**

We searched the PubMed, Embase, and CENTRAL databases for articles published prior to April 2020 to find observational studies of individuals with concurrent asthma and diabetes that compared the risk of asthma exacerbation between metformin users and nonusers. Two researchers separately screened the studies, extracted data, and evaluated the risk of bias. The primary outcome was the adjusted risk of asthma exacerbation. The secondary outcomes were the adjusted risk of asthma-related hospitalization and emergency room visits. Review Manager was used for data analysis and plotting. *I*^2^ and *χ*^2^ tests were used to estimate heterogeneity. A random effects or fixed effects model was used depending on the heterogeneity. Odds ratios were calculated for dichotomous variables.

**Results:**

We included two studies with a total of 25252 patients. The pooled effect size showed that metformin was inversely associated with a risk of asthma exacerbation (OR = 0.65, 95% CI 0.28–1.48; *χ*^2^ = 5.42, *P*=0.02; *I*^2^ = 82%), asthma-related emergency department visits (OR = 0.81, 95% CI 0.74–0.89; *χ*^2^ = 0.36, *P*=0.55; *I*^2^ = 0%), and hospitalizations (OR = 0.43, 95% CI 0.14–1.29; *χ*^2^ = 4.01, *P*=0.05; *I*^2^ = 75%).

**Conclusion:**

This meta-analysis suggested that metformin decreased the risk of asthma-related emergency room visits for patients with concurrent asthma and diabetes. Metformin reduced the risk of asthma-related hospitalization and exacerbation but was not statistically significant. More randomized trials involving larger samples should be considered, and the mechanisms of these effects need to be fully elucidated.

## 1. Introduction

Asthma, a chronic inflammatory disease of the small airway characterized by recurring wheezing, reversible airflow obstruction, cough, and other symptoms [1], affects more than 300 million people worldwide [2]. The WHO estimates that 235 million people currently suffer from asthma [3]. In China, more than 40 million people are affected by asthma, and the number has been increasing rapidly [4]. Asthma is treatable, but there is no cure [3]. It is estimated that more than 50% of asthma patients do not have control of the disease. Life-threatening exacerbations affect morbidity and mortality [5]. Controller and reliever medications are the main treatments for asthma [6]. The use of single maintenance and reliever therapy (SMART) was reported to be an excellent strategy for decreasing the risk of asthma exacerbations [6]. In addition, a series of monoclonal antibodies emerged against conventional drugs [7, 8]. However, the efficacy and safety of these new agents are not conclusive, and their price is not affordable for everyone. Therefore, cheap and effective adjuvant drugs are desirable.

Diabetes mellitus (DM) is a group of metabolic disorders characterized by a high blood sugar level, and DM is a common comorbid condition among adult patients with asthma [9]. Recent epidemiological studies have shown that the incidence rate of asthma in patients with type 2 diabetes is low, as metformin reduces the risk of asthma in patients with type 2 DM [9, 10]. In patients with concurrent asthma and diabetes population, the risk of asthma exacerbation is lower for metformin users [11, 12]. However, the efficacy of metformin for treating asthma has not been conclusive due to a lack of studies. We performed a systematic review and meta-analysis on this subject. Conclusions from such an analysis may help determine whether to use metformin as an adjuvant therapy for asthma.

## 2. Methods

### 2.1. Protocol and Registration

The protocol of the present systematic review was registered in the international platform of registered systematic review and meta-analysis protocols (INPLASY, https://inplasy.com/) and was reported following PRISMA [13] (Preferred Reporting Items for Systematic Reviews and Meta-Analyses) guidelines. The registration number is INPLASY202040210, and the DOI number is 10.37766/inplasy2020.4.0210.

### 2.2. Search Strategy

An online search was conducted for original studies published prior to Mar 2020, in the PubMed (http://www.ncbi.nlm.nih.gov/pubmed), Embase (http://www.embase.com), and Cochrane Central Register of Controlled Trials (CENTRAL) (onlinelibrary.wiley.com/cochranelibrary/) databases using the following search terms: (metformin[Title/Abstract] OR glucophage[Title/Abstract]) AND (asthma[Title/Abstract]). The search was performed in accordance with the Cochrane Handbook (LW and CY). In addition, we searched the references of all retrieved articles and relevant reviews (QZ and WZ).

### 2.3. Study Selection

The inclusion criteria were as follows: (1) observational studies; (2) patients age>18 years; (3) examined the use of metformin in patients with concurrent asthma and diabetes; (4) the odds ratios (ORs) or hazard ratios (HRs) of asthma exacerbation were compared between metformin users and nonusers.

The exclusion criteria were as follows: (1) animal or cell research; (2) reviews, letters to the editor, or case reports; (3) duplicate articles.

Two authors independently checked the titles and abstracts (GJ and GL). If there were different opinions between the reviewers, another author (ZM) was consulted to reach a consensus as the third investigator.

### 2.4. Data Collection Process

Data were extracted from each selected study (LH and YH), including the name of the author, year of publication, geographical location, study design, case number of metformin user/nonuser, age of metformin user/nonuser, gender of metformin user/nonuser, inclusion and exclusion criteria, follow-up, outcomes, and statistical approaches. We followed the Preferred Reporting Items for Systematic Reviews and Meta-Analyses (PRISMA) guidelines [13]. The quality of individual records was assessed according to the Newcastle-Ottawa Scale [14].

### 2.5. Outcomes

The primary outcome was the adjusted risk of asthma exacerbation. The secondary outcomes were the adjusted risk of asthma-related hospitalization and emergency room visits.

### 2.6. Statistical Analysis

Review Manager software (version 5.3; Cochrane Collaboration, Oxford, UK) was used to estimate the risks of bias of the included studies, analyze data, and create plots (WQ, YC, and CY). The sensitivity analysis and publication bias test were performed using *R* language if enough original studies were included. *I*^2^ and *χ*^2^ tests were used to estimate heterogeneity. If *P* > 0.1 or *I*^2^ <40%, a fixed effects model was used for the analysis. If there was a high degree of heterogeneity, a random effects analysis was used. Odds ratios (ORs) were calculated for dichotomous variables.

## 3. Results

### 3.1. Study Description and Risk of Bias

**Figure 1:**
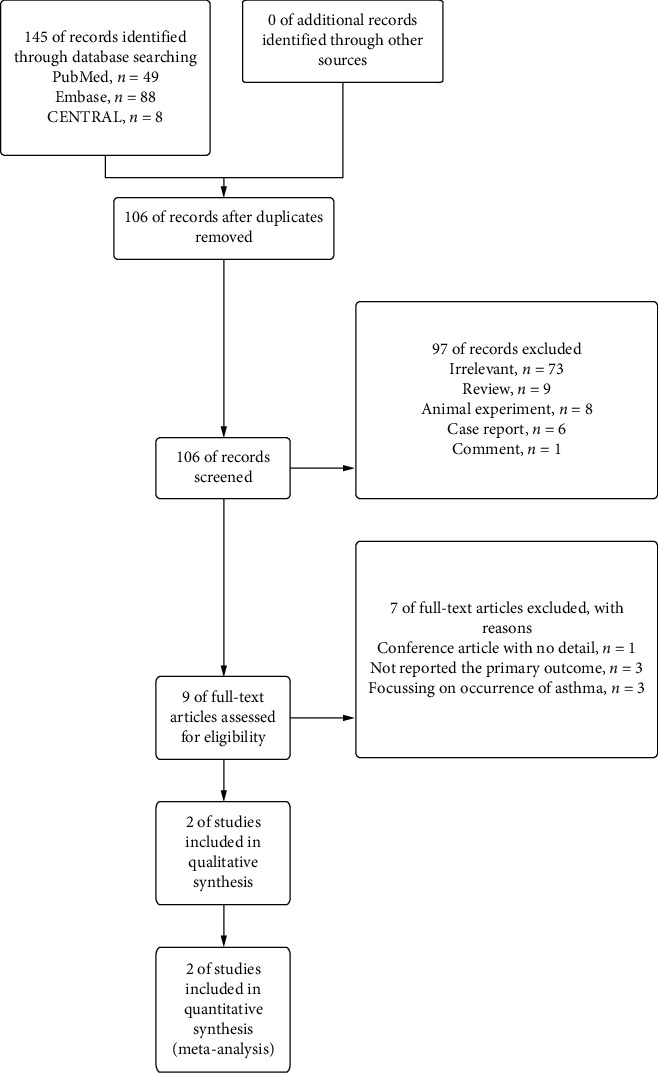
Flow diagram of the study selection.

By carrying out the search strategy mentioned above, a total of 106 articles were found after duplicated records were removed. After the title and the abstracts were screened, we downloaded the full texts of nine records, of which two were ultimately included in our analysis, including a total of 25252 participants. The details of the study selection process are shown in Figure 1. In total, two observational studies were included in the present meta-analysis, and the characteristics of the studies are shown in Table 1. The two trials were high-quality studies. The Newcastle-Ottawa Scale was used to evaluate the risk of bias for the observational studies (Table 2).

**Figure 2:**
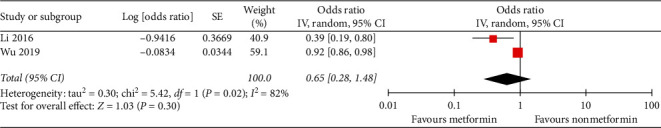
Metformin decreased the risk of asthma exacerbation in patients with concurrent asthma and diabetes without reaching the statistical significance.

Two observational studies reported the risk of exacerbation as the main outcome. The pooled effect showed that metformin decreased the risk of asthma exacerbation in patients with concurrent asthma and diabetes. There was a high level of heterogeneity, and the effect of metformin was not significant (OR = 0.65, 95% CI 0.28–1.48; *χ*^2^ = 5.42, *P*=0.02; *I*^2^ = 82%; Figure 2).

### 3.2. Secondary Outcomes

**Figure 3:**
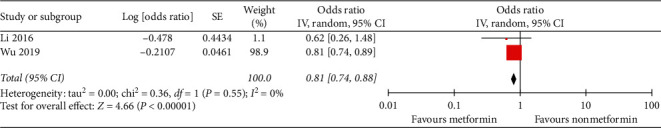
Metformin decreased the risk of asthma-related emergency room visits in patients with concurrent asthma and diabetes.

**Figure 4:**
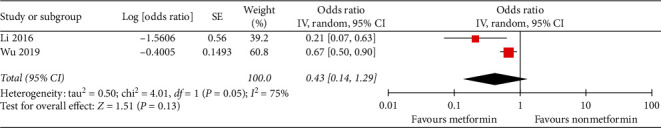
Metformin decreased the risk of asthma-related hospitalization in patients with concurrent asthma and diabetes, but this decrease was not statistically significant.

Two observational studies reported the risk of asthma-related hospitalization and emergency room visits. Metformin decreased the risk of asthma-related emergency room visits in patients with concurrent asthma and diabetes (OR = 0.81, 95% CI 0.74–0.89; *χ*^2^ = 0.36, *P*=0.55; *I*^2^ = 0%; Figure 3). Metformin decreased the risk of asthma-related hospitalization, but this finding was not statistically significant (OR = 0.43, 95% CI 0.14–1.29; *χ*^2^ = 4.01, *P*=0.05; *I*^2^ = 75%; Figure 4).

## 4. Discussion

In this meta-analysis of adults with asthma and type 2 DM, metformin use was inversely associated with a risk of asthma exacerbation, asthma-related emergency department visits, and hospitalizations, although the findings for the exacerbation and hospitalization were not statistically significant.

If we focus on the studies individually, metformin was shown to be associated with a decreased risk of the three outcomes in both studies. However, the nonsignificant results might be due to the heterogeneity. Both of the included studies analyzed overly heterogeneous population, with different average age points, inclusion and exclusion criteria, follow-up periods, and statistical methods. On the one hand, this made it impossible to draw definitive conclusions regarding the subject matter. As shown above, there was a high level of heterogeneity and the effect of metformin was not significant. On the other hand, the two research studies validated each other with different populations. To some extent, they are likely to have promoted the generalizability of the effect of metformin on asthma with type 2 DM. We expected that more homogeneous studies will soon emerge in the future, so that researchers could draw more definitive conclusions. Type 2 DM and asthma are both extremely heterogeneous diseases in terms of pathogenesis and clinical characteristics. Metabolism-related asthma is not the same as allergic asthma, and eosinophilic and neutrophilic asthma may differ in the response of metformin. It is regrettable that the included two studies did not report the subgroup data of the phenotypes regarding asthma or type 2 DM.

Drug therapies used in the group that did not take metformin were mentioned in the baseline characteristics of the two cohorts. In one article [12], there were no difference of the percentage of drugs use between two groups for asthma and type 2 DM. However, in another publication [11], there were different proportions of SABA, corticosteroid, methylxanthine, meglitinide, and insulin users between observational and control groups. Insulin has been reported to be associated with increased risk of asthma [10]. Different corticosteroid user percentages indicated that severity between two groups may not be the same. This could represent one of the methodological biases that account for the nonsignificance of the results and the heterogeneity. However, these findings suggest a potential application for metformin in decreasing the risk of severe asthma exacerbations.

Metformin, a widely used medication for type 2 DM, has been shown to be associated with a decreased risk of chronic lower respiratory disease mortality, and no similar effect has been found for other antidiabetic medications [15]. Several studies have reported that metformin use reduces the risk of asthma in patients with diabetes [9, 10], but the mechanism of this effect is not fully understood. Several possible mechanisms are discussed as follows.

Obesity, metabolic abnormalities, type 2 DM, and asthma are linked to each other. Obesity is a major risk factor for both type 2 DM and asthma [16]. Obese patients with asthma tend to have more symptoms and exacerbation frequency, as well as decreased response to asthma treatments [17]. Metabolic abnormalities, especially insulin resistance, frequently coexist with obesity, which have been proved to be associated with asthma and pulmonary function decrease [18, 19]. Insulin resistance is likely to play an important role in the pathophysiology of asthma [20]. There is more significant association between obesity and asthma if insulin resistance exists [21]. Fortunately, metformin is an effective agent in improving metabolism, weight loss, and glucose reduction by alleviating insulin resistance. Insulin resistance is the common point in the problems listed above, which would be alleviated effectively by metformin.

Metformin is also associated with changes in many asthma-related cells and cytokines. Ig-E and aryl hydrocarbon receptor-mediated mast cell activation could be inhibited by metformin in vitro and in vivo [22]. Metformin downregulated the expression levels of inflammatory cytokines (IL-1*β*, IL-4, and IL-6 and TNF-*α*) in intracerebral hemorrhage model rats [23]. Treatment with metformin showed ability to attenuate upregulation of IL-4-DUOX2 pathway and other pathological damages to the lung after exposure to a high dose of ionizing radiation [24]. In an animal experiment, metformin increased the ratio of Treg/Th17 cells in mice [25]. As we all know, molecules such as IL-4, IL-6, TNF-*α*, Ig-E, and Th17cell played key roles in the pathological process of asthma. Effects of these molecules and cells may be the vital mechanisms of metformin on asthma.

AMP-activated protein kinase (AMPK) is an enzyme that plays an important role in the regulation of insulin signaling and glucose metabolism [26], which could reduce inflammatory responses and airway remodeling in the respiratory system [27, 28]. Metformin activates AMPK, which suppresses glycolysis in immune cells and suppresses cytokine production in vitro and in vivo [29]. Metformin has been shown to attenuate allergic eosinophilic airway inflammation in obese mice and restore levels of AMPK in lung tissue [30]. It may also inhibit airway smooth muscle cell proliferation through AMPK-dependent pathways [31]. More than one article reported that metformin reduces airway inflammation, fibrosis, and remodeling by activation of AMP-activated protein kinases and inhibition of mTOR signaling [28, 32]. It seems that AMPK is the common ground of asthma and diabetes, which could be activated by metformin.

Other possible mechanisms were also mentioned in the literature. It was found that the p38MAPK signal transduction pathway increased in both type 1 and type 2 diabetes [33], which has been shown to induce an anti-inflammatory response in vascular smooth muscle cells in diabetic mice [34]. Metformin, as an inhibitor of p38MAPK, may act as a potential treatment for asthma. LPS-induced bronchial epithelial cell injury could be ameliorated by metformin via NF-*κ*B signaling suppression [35]. Metformin induced decreases in the VEGFa level in the airway in obese mice to alleviate airway remodeling [36].

These examples may be mechanisms by which metformin interferes with asthma. In summary, the discovery of the mechanisms of metformin on respiratory disease is limited, and more basic research should be carried out.

Several limitations should be mentioned. Only two studies were ultimately included in this meta-analysis, which is a low number. Therefore, subgroup analysis, sensitivity analysis, and publication bias evaluation could not be conducted. In the absence of high-quality randomized controlled trials, the reliability of the conclusions of this review should be verified in the future.

Finally, we expect that clinical trials assessing the use of metformin in patients with asthma with or without DM should be performed.

## 5. Conclusion

In summary, this meta-analysis suggested that metformin decreased the risk of asthma-related emergency room visits for patients with concurrent asthma and diabetes. Metformin reduced the risk of asthma-related hospitalization and exacerbation, but these findings were not statistically significant. These findings suggest a potential role for metformin in the respiratory system. Prospective cohort or randomized trials should be considered, and the underlying mechanisms of these effects need to be fully elucidated.

## Figures and Tables

**Table 1:** Characteristics of the two eligible studies and their participants.

First author (year)	Region	Design	Number of MU/MNU	Age MU/MNU	Gender F(M) MU/MNU	Inclusion and exclusion criteria	Follow-up	Outcomes	Statistical methods
Li [11], 2016	Taiwan Province, China	Retrospective cohort	444/888	64 (10.1)/64 (10.1)	268 (176)/536 (352)	Inclusion criteria: (1) aged ≥18 years with concurrent asthma and diabetes; (2) patients who have had at least one inpatient or two outpatient diagnoses of asthma and diabetes during the enrollment period; (3) patients who had at least one prescription for asthma and diabetes medication during the enrollment period; (4) matched patients' date of the asthma and diabetes diagnosis must be earlier than the index date	3 years	Adjusted odds ratio of asthma hospitalization, emergency room visit, and exacerbation	Multivariable logistic regression
						Exclusion criteria: (1) patients who had a metformin prescription within 1 year before the index date; (2) patients if they had been diagnosed with COPD, any respiratory tract cancer, or bronchiectasis during the preindex period; (3) patients if they had an asthma-related hospitalization or emergency room visit during the preindex period; (4) patients with invalid or missing information of age, gender, diagnosis codes, medication prescriptions, and enrollment records			
Wu [12], 2019	50 states of USA	Retrospective cohort	11960/11960	51.9 (9.3)/51.9 (9.9)	7894 (4066)/7902 (4058)	Inclusion criteria: (1) adult participants (age 18 or older) with both asthma and diabetes; (2) patients who had at least two compatible outpatient codes or one inpatient code during enrollment; (3) qualifying outpatient claims to be within one year	6 years	Adjusted odds ratio of asthma hospitalization, emergency room visit, exacerbation, and corticosteroid use	Cox proportional hazards model
						Exclusion criteria: (1) individuals with any diagnosis of chronic obstructive pulmonary disease, bronchiectasis, or interstitial lung disease; (2) those with a contraindication for metformin use, type I diabetes, and a rheumatologic condition that may require systemic corticosteroids for symptoms unrelated to asthma			

MU: metformin user; MNU: metformin nonuser.

**Table 2:** Risk of bias of included cohort studies.

	Selection				Comparability		Outcome	
	(1) Representativeness of the exposed cohort	(2) Selection of the nonexposed cohort	(3) Ascertainment of exposure	(4) Demonstration that outcome of interest was not present at start of study	(1) Comparability of cohorts on the basis of the design or analysis	(1) Assessment of outcome	(2) Was follow-up long enough for outcomes to occur?	3) Adequacy of follow-up of cohorts

Li [11]	1	1	1	1	1	1	1	1
Wu [12]	1	1	1	1	1	1	1	1

Primary outcomes are given.
